# FisherMP: fully parallel algorithm for detecting combinatorial motifs from large ChIP-seq datasets

**DOI:** 10.1093/dnares/dsz004

**Published:** 2019-04-08

**Authors:** Shaoqiang Zhang, Ying Liang, Xiangyun Wang, Zhengchang Su, Yong Chen

**Affiliations:** 1 College of Computer and Information Engineering, Tianjin Normal University, Tianjin, China; 2 Department of Bioinformatics and Genomics, the University of North Carolina at Charlotte, NC, USA; 3 Department of Biological Sciences, Center for Systems Biology, the University of Texas at Dallas, Richardson, TX, USA

**Keywords:** combinatorial motifs, parallel algorithm, ChIP-seq

## Abstract

Detecting binding motifs of combinatorial transcription factors (TFs) from chromatin immunoprecipitation sequencing (ChIP-seq) experiments is an important and challenging computational problem for understanding gene regulations. Although a number of motif-finding algorithms have been presented, most are either time consuming or have sub-optimal accuracy for processing large-scale datasets. In this article, we present a fully parallelized algorithm for detecting combinatorial motifs from ChIP-seq datasets by using Fisher combined method and OpenMP parallel design. Large scale validations on both synthetic data and 350 ChIP-seq datasets from the ENCODE database showed that FisherMP has not only super speeds on large datasets, but also has high accuracy when compared with multiple popular methods. By using FisherMP, we successfully detected combinatorial motifs of CTCF, YY1, MAZ, STAT3 and USF2 in chromosome X, suggesting that they are functional co-players in gene regulation and chromosomal organization. Integrative and statistical analysis of these TF-binding peaks clearly demonstrate that they are not only highly coordinated with each other, but that they are also correlated with histone modifications. FisherMP can be applied for integrative analysis of binding motifs and for predicting *cis*-regulatory modules from a large number of ChIP-seq datasets.

## 1. Introduction

In the past two decades, the motif-finding problem has been an important issue in sequence feature recognition. A motif represents a set of binding sites recognized by a transcription factor (TF). Based on co-regulation of genes and phylogenetic footprinting of TFs, TF motifs can be discovered from a set of upstream non-coding DNA sequences of co-regulated or orthologous genes. Many motif-finding algorithms have been developed in the past two decades including MEME,^1^ BioProspector,^2^ Weeder,^3^ MotifClick^4^ and so on. These traditional motif finders search for over-represented segments with higher significance than non-binding segments. In the last decade, some new experimental techniques such as chromatin immunoprecipitation sequencing (ChIP)-chip and ChIP-seq have been developed to locate TF-binding sites.^5^^,^^6^ By using these technologies, many motif datasets have been generated for a variety of model organisms. For example, there are Lee/Harbison ChIP-chip and ChIP-seq datasets for *Saccharomyces cerevisiae,*^7^^,^^8^*Caenorhabditis elegans* and *Drosophila melanogaster* in the modENCODE project^9^^,^^10^ as well as human ChIP-seq datasets in the ENCODE project.^11^ One ChIP-seq experiment for a TF may produce millions of binding peak sequences by running peak-calling tools, such as MACS2.^12^^,^^13^ In the ENCODE database, over 60% of binding peak datasets have more than 10,000 sequences, the largest of which can have a million sequences. However, many of them include false positives due to binding peaks introduced through the mediation of protein complexes, reducing the precision of motif-finding tools. A further challenge is to find specific combinations of binding sites of potential co-regulatory TFs, which are widely observed for *cis*-acting transcriptional regulatory elements in mammalian genomes.^14^^,^^15^ However, most popular tools such as MEME,^1^ GLAM2,^16^ W-ChIPMotifs^17^ and XXmotif^18^^,^^19^ can only model a single motif at a time and do not detect alternative binding motifs of co-factors.^20^ Therefore, it is critical to develop a fast and efficient method for processing the increasing ChIP-seq data to discover binding motifs as well as their combinations of potential co-factors.

To find motifs from ChIP-seq datasets, many motif-finding tools, including DREME,^21^ HOMER,^22^ Amadeus,^23^ Trawler,^24^ motifRG,^25^ XXmotif,^18^^,^^19^ DECOD^26^ and FastMotif,^27^ have been specially designed. The vast majority of these new motif finders used a ‘discriminative’ strategy, meaning that the motif patterns should discriminate between a foreground sequence dataset and a background sequence dataset. However, they usually took a lot of computing time to find binding motifs of targeted TFs, especially for those that have millions of binding peaks called from very deeply sequenced data. In order to speed up the motif-finding procedure, these algorithms adopted some approximation schemes that include restrictions on search space (e.g. HOMER, XXmotif), the *P*-value estimation by approximation formula (e.g. DREME), replacing position weight matrices (PWMs) by IUPAC characters^28^ (e.g. DREME and motifRG), or replacing word-based methods by probabilistic sampling (e.g. FastMotif). Although several tools such as DREME, HOMER, MotifRG and FastMotif have been evaluated to be faster than other tools according to recent comparative analysis,^21^^,^^27^^,^^29^ they still consume a lot of time on large-scale datasets. Furthermore, these programmes are extremely slow for detecting the combinatorial motifs from large-scale ChIP-seq datasets that often occur in genomes.^30^ Parallel computing is a promising strategy for large data computation. HOMER and MotifRG have the option to set the number of processes, but they are still not fully parallel algorithms. Actually, HOMER is designed for a fixed motif length. The multithreading technique used in HOMER is called ‘Pthread’, which is a thread API built in Linux. When calculating *k* multiple motif lengths, *k* threads are called to run the same whole HOMER programme simultaneously. When the number of considered motif lengths is less than the number of available threads, HOMER cannot use the full computational resource. MotifRG is an R package that directly employs the parallel package of Bioconductor to call multiple cores. Although the iterations of enumerating and evaluating candidate motifs were performed in parallel, the other steps such as refining the top motifs were not parallelized. Furthermore, after getting the refined motifs, MotifRG masks all of their occurrences and repeats the same process to find next motifs sequentially, which can be further optimized by well-designed parallel strategies. Therefore, these computational tools only partially performed parallelization techniques at one or limited steps. As far as we know, only MEME and Gibbs Sampler-based algorithms have been parallelized with CUDA,^31–33^ and there is no specialized parallel algorithm for discriminative motif discovery in ChIP-seq data. Since these word-based algorithms should determine whether each k-mer in a sequence set is highly conservative (or discriminative), we can parallelize this step for all of the k-mers with different lengths.

Based on these observations, a fully parallel algorithm called FisherMP is presented to predict the short and core-binding sites of a TF from its large-scale-binding peaks identified via ChIP-seq. FisherMP is ultra-fast and can discover genome-wide motifs from a large number of ChIP-seq datasets. In particular, the FisherMP algorithm can find the motifs of a TF’s co-factors from its binding peaks. Our analysis on CTCF ChIP-seq datasets of human chromosome X revealed the high correlation of CTCF and YY1 motifs. The combinatorial motifs of CTCF and YY1 were further confirmed on multiple human cell lines and integrative analysis of histone modification signals, indicating that they are functional co-players.

## 2. Materials and methods

### 2.1. Motivation and methodology overview

FisherMP is a ‘discriminative’ motif-finding programme which requires two input data files; one is the (foreground) data file containing motifs to be sought, and the other is an explicitly collected background data file. FisherMP uses Fisher’s exact test to calculate *P*-values of motifs. In FisherMP, the regular expression based on IUPAC symbols used in DREME^21^ is discarded, and the motifs are constructed directly based on word conservation. Furthermore, in order to obtain the corrected *P*-value of each motif, FisherMP utilizes a hash of arrays to store the indices of all possible sequences for each existing word instead of estimating the number of sequences containing each word of a motif as in DREME. Unlike DREME and MotifRG, which directly merge IUPAC motifs, FisherMP merges two motifs into a bigger one if they reach a specified high similarity score. The motif similarity metric used in FisherMP is SPIC,^34^ which is constructed by using information contents, PWMs and position frequency matrices (PFMs) of motifs. The parallel implementation of FisherMP is completely based on OpenMP, which is a standard API for portable shared memory parallel programming in C/C++ and is supported by the GNU Compiler Collection (GCC).

### 2.2. The FisherMP algorithm with parallel computing design

**Figure 1:**
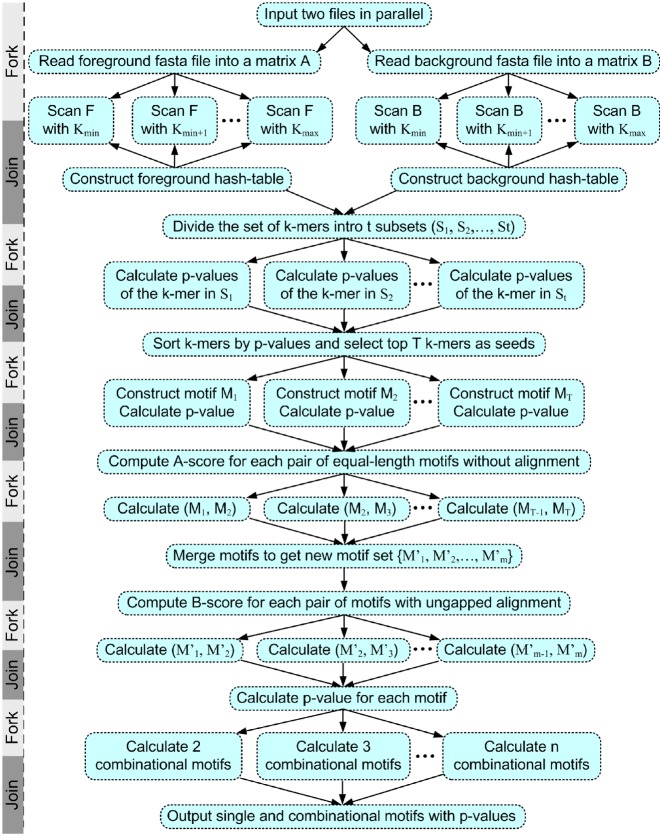
Flowchart of FisherMP with parallel computing design.

The (foreground) input file should be a set of ChIP-seq-binding peak regions from a TF ChIP-seq experiment which can be identified by a peak-calling algorithm. The (background) negative sequence set should either be a similar dataset from a different ChIP-seq experiment or a set of randomly generated sequences based on the distribution of bases in the foreground file. In order to parallelize the programme, FisherMP employs six ‘fork-join’ structures. The flowchart of FisherMP with parallel design is shown in Fig. 1.

**Step 1:** *Reading the input files*. If there is a background file, two threads are called to synchronously read the foreground and background files into two matrices (i.e. vectors of vectors) *F* and *B* of nucleotide bases, respectively. In *F* and *B*, each sequence in the input files corresponds to a row vector of base pairs. In addition, for the later construction of PWMs, the frequency of each nucleotide is counted at the same time. If there is not a background file, the matrix *B* is generated randomly based on the frequencies of nucleotides with the same size of *F*.

**Step 2:** *The first ‘fork-join’ structure*. *F* and *B* are scanned with different window widths *k* in parallel to count all of their k-mers and construct two hash maps. Multiple threads are called for different window widths *k* and the two different matrices. Ideally, the optimal number of threads is 2(*k*_max_–*k*_min_+1) if *k*=*k*_min_, *k*_min_+1, …, *k*_max_. The hash map of *F* (or *B*) stores all the k-mers and the index set of the foreground (or background) sequences in which they are located. More specifically, a k-mer associated with its reverse complement is stored as a key of the hash map, and the corresponding value of the key is the index array of sequences containing the k-mer or its reverse complement.

**Step 3:** *The second ‘fork-join’ structure*. *P*-values of all k-mers based on Fisher’s exact test are calculated in parallel. From the above two hash maps, for each k-mer and its reverse complement, the numbers of sequences containing either of them in *F* and *B* can be directly calculated and used to model the Fisher’s exact test by constructing a contingency table (Supplementary Section S1 and Supplementary Table S1). In the Fisher’s exact test, the p-value of a k-mer is given by a hypergeometric distribution, which can be approximated by the Stirling formula^35^ (Supplementary Section S1). The *P*-values of different k-mers can be calculated in different threads because there is no data interchange among the threads. If *t* threads are called, all the k-mers in *F* can be divided into *t* groups evenly. As a result, all the k-mers in *F* and their corresponding *P*-values are stored in a hash map (for a k-mer and its reverse complement, only one is stored in the hash map).

**Step 4:** *Motif seeding and generating in the third ‘fork-join’ structure*. An array *A* of k-mers is formed by sorting all k-mers in *F* by *P*-values in numerically ascending order and then deleting the k-mers with *P*-values > 0.05. The top *m* (the default is 100) k-mers in the array *A* are selected as motif seeds. For a motif seed *s*, a *primitive motif M* is constructed as follows:

- The motif *M* is initially set as{s}, and the *P*-value of *M* is set as the *P*-value of *s*.
- Starting from the last k-mer added to *M* in the array *A*, select the top k-mer *t* (or its reverse complement) in the following queue of *A* as a *candidate element* for *M* if (a) only one position of *t* (or its reverse complement) is different than one of the k-mers in *M* and (b) at most *α* percent (the default is 30%) positions are not conserved among *t* (or its reverse complement) and all the k-mers in *M*. If there is no such a candidate element, return *M* and stop.
- Calculate the *P*-value of M∪{t}. If the *P*-value of M∪{t} is less than *β* (the default value is 0.05), *t* is added into *M* and go to (b), else return *M* and stop.

Notice that for each k-mer the foreground and background indices of sequences containing it or its reverse complement have been stored in two hash maps respectively built in Step 2. Therefore, for a motif *M* composed of *u* k-mers, the union of foreground (or background) sequence index sets of the *u* k-mers forms the motif’s foreground (or background) sequence index array, whose size is used to calculate the *P*-value of *M*. In the end, for each primitive motif *M*, the PFM, PWM and the position information contents (PICs), which will be used to calculate motif similarity in the next step, are generated based on the counts of k-mers in *M* and the frequency of each nucleotide in *F* (Supplementary Section S2). Clearly, the primitive motif construction process can be conducted in parallel for each seed. If *t* threads are called, these seeds can be split into *t* groups evenly.

**Step 5:** *Computing motif similarities in the fourth ‘fork-join’ structure*. The SPIC metric, which was shown to have the best performance among several metrics,^34^ is used to calculate the similarity between each pair of primitive motifs. For each pair of primitive motifs with the same motif length, the SPIC metric without alignment is used to calculate their similarity score which is called their “*A-score*” (Supplementary Section S2).

For each pair of equal-length primitive motifs *M_i_* and *M_j_* where *i* < *j*, the PFM, PWM and PICs of *M_j_* are first transferred into their reverse complementary forms, then two A-scores between *M_i_* and *M_j_* and between *M_i_* and the reverse complement of *M_j_* are separately calculated, and the larger one is selected as their final similarity score. Notice that all pairs of motifs can be evenly distributed to all threads because the similarity calculation between a pair of motifs does not require the information of any other motifs.

**Step 6:** *Motif merging*. If a set of equal-length primitive motifs have high A-scores among one another, then they are merged into a new motif and the *P*-value of the new motif is also calculated.

**Step 7:** *Computing merged motif similarities in the fifth ‘fork-join’ structure*. For each pair of merged motifs, the SPIC metric with ungapped alignments is used to calculate their similarity score which is called their ‘*B-score*’ (Supplementary Section S2). The calculation procedure of B-score is exactly the same as that of A-score in Step 5.

**Step 8:** *Motif deleting and sorting*. If two motifs share the highest similarity B-score, the motif with the higher *P*-value is removed from the motif set. The refinement is repeated until no pair of motifs has a higher B-score than the given threshold.

**Step 9:** *Computing combinatorial motifs in the sixth ‘fork-join’ structure*. With the single motif and its *P*-value available, we further calculate the *P*-value for the combinations of 2, 3, …, n motifs (the default is 2) by using a Fisher combined probability test^36^^,^^37^ (Supplementary Section S3). Finally, the single and combined motifs are sorted by *P*-values in ascending order.

Note that the two steps of scanning all the k-mers in the foreground and background sequences and calculating their *P*-values are the most time consuming in all ‘word-based’ algorithms. In FisherMP, two ‘fork-join’ structures are designed to perfectly parallelize the two steps.

### 2.3. Threshold settings in FisherMP

In order to find real motifs accurately, the parameters in the FisherMP algorithm should be set scientifically. At the stage of generating the primitive motifs in Step 4, it is necessary to set the ratio *α* of non-conserved positions in each primitive motif. For this purpose, the PICs of the 1,404 CORE PFM profiles in the JASPAR2018 database were calculated (http://jaspar.genereg.net/downloads/). If the information content of a position in a motif is <1 (note that any PIC’s value is between 0 and 2), then the position is considered relatively non-conserved. The percentage of relatively non-conserved positions was counted for each CORE PFM in JASPAR. We set the parameter *α* as 30% in FisherMP since 74% of the 1,404 motif profiles each contain at most 30% relatively non-conserved positions (Supplementary, Section S4 and Supplementary Fig. S1A).

Furthermore, the threshold settings of the A-score and B-score used in FisherMP are based on the distribution of similarity between two sub-motifs randomly split from each known motif in JASPAR. For each motif in the JASPAR2018 CORE database, it was split into two sub-motifs randomly and the A- and B-score were calculated between the two sub-motifs. This process was repeated 10 times. For each pair of different motifs in JASPAR, the B-score was calculated, and A-score as well if the two motifs had the same length. The distributions of A-scores and B-scores between two different motifs and pairs of sub-motifs (of the same motif) are separately plotted. For the majority of the real motifs, the similarity A-scores and B-scores between two sub-motifs of the same motif were around 0.8, while the similarity scores between different motifs were generally around 0 (Supplementary Section S4and Supplementary Fig. S1B). In the step of motif merging, the threshold of A-score was set to a relatively high value 0.7 in order to reduce the chance of merging primitive motifs which do not belong to the same real motif. After the motif merging step, we found that there were very few pairs of motifs with B-scores > 0.7. Thus, in the following step of redundant motif deleting, the B-score threshold was set to a relatively high value of 0.6 in order to reduce the probability of deleting a non-redundant motif.

### 2.4. Datasets used

The datasets of TF ChIP-seq uniform peaks used in the paper were generated by the ENCODE project. There are 690 binding peak files available in the UCSC genome browser (http://genome.ucsc.edu/cgi-bin/hgFileUi? db=hg19&g=wgEncodeAwgTfbsUniform). To assess FisherMP on real data, a total of 350 ENCODE ChIP-seq-binding peak datasets belonging to 51 TFs which have known (literature) motif profiles were selected from the Kheradpour and Kellis’s collection (http://compbio.mit.edu/encode-motifs/).^38^ The summarization of the ENCODE datasets, known motifs and discovered motifs used in the paper are shown in Supplementary Table S2. The binding peak file of ER in MCF-7 cells was downloaded from GSE19013 (https://www.ncbi.nlm.nih.gov/geo/query/acc.cgi? acc=GSE19013).^39^

### 2.5. Recovering motif pairs

The known TF combinations were downloaded from TcoF database^40^ (latest version, March 2018). In our 51 selected TF ChIP-seq datasets, 21 TFs have known combinatorial motifs including 30 combinatorial motif pairs. FisherMP was run on these 21 TF ChIP-seq datasets to detect not only the TF motifs themselves but also their combinatorial motifs. To reduce bias, the ChIP-seq datasets for each pair of cooperative TFs were selected from the same cell line as much as possible.

### 2.6. Performance assessment

In order to verify whether a predicted motif is identical to a known motif, the SPIC metric with ungapped alignments (i.e. the B-score) is employed to calculate their similarity.^34^ The SPIC metric is extremely robust because it considers sequence position in additional to identity. The SPIC metric has been optimized to have the best performance for separating true motifs from putative motifs after comparing it to seven other motif similarity metrics including Pearson correlation coefficient, average log-likelihood ratio, sum of squared distances, asymptotic covariance, *P*-value of Chi-square, average Kullback-Leibler and k-mer frequency vector.^34^^,^^41^

To test the accuracy of a predicted motif for each algorithm, for a binding peak dataset of a TF, the similarity B-score was calculated between its predicted motif and each known motif profile of the TF, and only the highest similarity score was taken as the final similarity between the predicted motif and the real motif of the TF. If a TF *TF_i_* has *m_i_* known motif profiles $\left\{M_{i1},M_{i2},\ldots ,M_{im_{i}}\right\}$ and *d_i_* datasets $\left\{D_{i1},D_{i2},\ldots ,D_{id_{i}}\right\}$, supposing that $P_{\mathrm{ij}}$ is the predicted motif output by a motif-finding programme for the dataset $D_{\mathrm{ij}}$, we say that $P_{\mathrm{ij}}$ is a real motif discovered correctly by the programme if ${\text{maz}}_{t=1}^{m_{i}}\left\{\mathrm{Bscore}\left(P_{\mathrm{ij}},M_{\mathrm{it}}\right)\right\}$ is greater than a similarity threshold *γ*. For all the 350 datasets of 51 TFs, the prediction accuracy (PA) of a motif-finding programme can be defined as

(1) $$\mathrm{PA}=\frac{\sum_{i=1}^{51}\sum_{j=1}^{d_{i}}\mathrm{sgn}\left(\max_{t=1}^{m_{i}}\{\mathrm{Bscore}(P_{\mathrm{ij}},M_{\mathrm{it}})\}>\gamma \right)}{\sum_{i=1}^{51}d_{i}},$$

where sgn (·) is an indicator function which returns 1 if the argument is true and 0 otherwise.

Note that besides the known literature motif profiles; there are often multiple discovered motifs for each of the 51 TFs in the Kheradpour and Kellis’s collection (Supplementary Section S5 and Supplementary Table S2). For convenience, we call these discovered motifs ‘*KK motifs*’. These KK motifs were collected from the top 10 most enriched motifs (excluding duplicates) discovered by multiple motif-finding tools for each TF group.^38^ A large part of these KK motifs are likely to be the true motifs of these TFs and their co-factors. Each tool was evaluated by counting the number of recovered KK motifs in its top 10 predicted motifs in each dataset.

If *TF_i_* has *k_i_* KK motifs and *d_i_* datasets, for each dataset *D_ij_*, suppose that *R_ij_* of the *k_i_* KK motifs are recovered by the top 10 predicted motifs output by a motif-finding tool. We said a KK motif is ‘recovered’ if there is a motif in the top 10 predicted motifs such that the similarity B-score between the KK motif and the predicted motif is greater than a similarity threshold *γ*. Then the average recovered rate (ARR) for all datasets is defined as

(2) $$\text{ARR}=\frac{\sum_{i=1}^{51}\sum_{j=1}^{d_{i}}R_{\mathrm{ij}}}{\sum_{i=1}^{51}d_{i}k_{i}}$$

We also calculated the ROC curve by using the methods provided in a previous study.^42^ In detail, 10 motifs of lengths from 5 to 10 were picked out from JASPAR, and then each of the 10 motifs was separately implanted into each of 10 datasets to produce 100 synthetic-binding peak files. The *i*th dataset consists of 1,000⋅i sequences of length 1,000 (*i* = 1, 2,…, 10). We ran the seven motif discovery algorithms to output top 20 motifs on each of the 100 synthetic peak files. With the rank of output motifs increasing, we plotted the receiver operating characteristic (ROC) curves and the areas under these curves (AUCs) for seven algorithms.

In order to test the computational speeds of the algorithm for input datasets with different sizes, we conducted experiments with both synthetic data and real data. The length of each sequence in any synthetic dataset was set to 1,000 base-pairs (bp). The dataset sizes were arranged from 0.1 to 1 Mb in steps of 0.1 Mb, and from 1 to 10 Mb in steps of 1 Mb. For each setting of dataset size, 10 synthetic datasets were generated randomly based on a three-order Markov model on ENCODE-binding peak datasets, and each element of a motif were randomly picked out and inserted into each synthetic sequence. For each dataset size, 10 datasets of real data were also extracted separately from the top sequences of 10 different files which were picked up from the 690 ENCODE-binding peak files. As a result, for each motif-finding programme, the average running time was reported for each synthetic (or real) dataset size.

### 2.7. Programme selection and parameter settings

Based on the previous literature on the comparison of motif-finding algorithms, the six new tools, DREME, HOMER, MotifRG, XXmotif, FastMotif and DECOD perform better than others on discovering true motifs.^18^^,^^19^^,^^21^^,^^26^^,^^27^^,^^29^ Therefore, these top tools were selected to compare the accuracy with that of FisherMP. In order to compare the computational speeds of different algorithms, four relatively fast tools, HOMER, DREME, MotifRG and FastMotif, were selected from these new tools. Note that XXmotif and DECOD were discarded for comparing computational speeds because their running times are significantly longer than the others. WSMD was also not used for comparison because it requires a commercial payment component, claims to be slower than HOMER, and is based on weak supervised learning and thus has compromised accuracy.

To maximize experimental integrity, the parameter settings of these tools were kept as consistent as possible. The maximum number of motifs to find was set to 10 (e.g. FisherMP, DREME, MotifRG and DECOD). If there was no such option to set motif number in a motif-finding tool (e.g. HOMER, XXmotif and FastMotif), then the top 10 output motifs were used for performance comparison. If the motif length range could be set in a tool, then the lengths of output motifs were set from 5 to 10 (e.g. FisherMP, DREME, HOMER and MotifRG). Otherwise, the motif length was fixed to be 10 (e.g. DECOD). All the background negative sequences used in these tools were generated by themselves except DECOD, whose background sequences were generated by shuffling the corresponding foreground sequences. Note that multiple processer cores can be called by FisherMP, HOMER and MotifRG. The number of threads was set to four in HOMER and MotifRG due to the popularity of quad-core processors. Four and six threads were tested separately for FisherMP. Besides the above settings, the motif search was double-stranded if possible, the *P*-value cut-off was 0.05, and all other settings were set by default. The specific commands of the seven tools used in the paper are described in the last section of the Supplementary Material. All of the experiments were done on a 64-bit Linux server with two 8-core CPUs (Intel Xeon E5 2.1 GHz).

## 3. Results

### 3.1. High performance of FisherMP for finding motifs on human ChIP datasets

**Figure 2:**
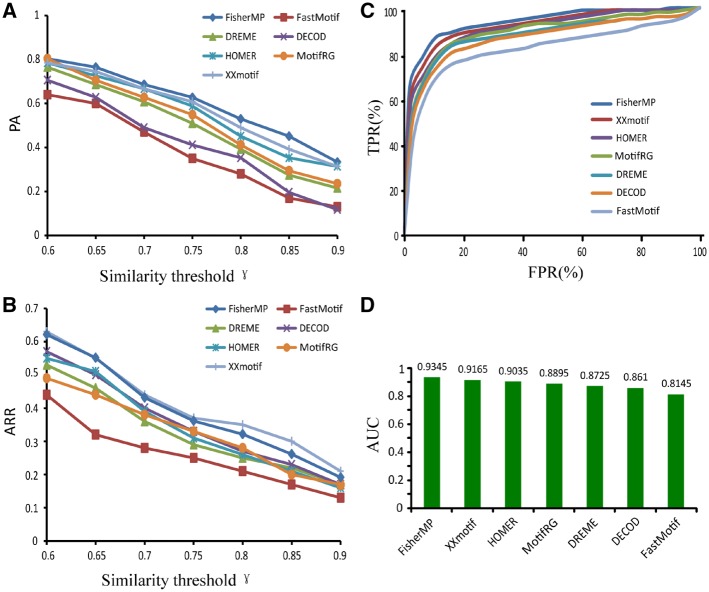
Performance comparison of FisherMP with six other motif-finding methods. (A) The distributions of PA for seven motif-finding programmes under different similarity thresholds. (B) The distributions of ARR under different similarity thresholds. (C) The ROC curves of seven programmes. FPR and TPR. (D) The AUC scores of seven programmes.

To evaluate the performance of FisherMP for finding TF motifs, we ran it on all 350 ChIP-seq experiments of 51 TFs that were downloaded from the ENCODE project. The validations on genome-wide ChIP experiments for TFs of multiple cell lines provided unbiased performances in real applications. Since the predicted motifs are defined by their consensus sequences, we considered the real motif of a TF as recalled if its similarity to its predicted motifs was larger than a certain similarity threshold. We used two criteria: the PA and ARR. For these 51 TFs, the PA was defined as proportion of recalled motifs among all 350 datasets. We found FisherMP achieved as high as 80% for the similarity threshold of 0.6 (Fig. 2A). When ranging similarity threshold from 0.6 to 0.9, the PAs of FisherMP were decreasing, suggesting that the predicted consensus motifs are different than the real motifs in some of datasets.

In the Kheradpour and Kellis’s collection,^38^ many motifs are likely to be the true motifs of these TFs and their co-factors, named KK motifs. We collected the KK motifs of our studied 51 TFs and defined a KK motif as ‘recovered’ if the top 10 predicted motifs contained a motif such that the similarity between the KK motif and the predicted motif was greater than a similarity threshold. Then the ARR was defined as averaged proportions of each TF’s recovered KK motifs among all 350 datasets. Consistent with the previous PR results, we found FisherMP achieved an ARR as high as 62% for the similarity threshold of 0.6 (Fig. 2B). Taken together, these cross TFs and cross cells validations indicate FisherMP can achieve accurate performance not only for single motifs, but also for multiple alternative motifs of a TF.

### 3.2. Comparing FisherMP with other methods

We compared FisherMP with six other motif-finding tools on these 350 ChIP-seq datasets. All methods were separately executed, and the PA and ARR values for each programme were calculated for different motif similarity thresholds. As shown in Fig. 2A, FisherMP achieved the best PR score of 80% for a similarity threshold of 0.6. Although PR scores were decreasing when similarity thresholds increased, FisherMP kept the optimum PR scores among these methods. For the ARR scores, we found FisherMP and XXmotif were better than other methods. Specifically, FisherMP can achieve an ARR score of 63% when similarity thresholds ranged from 0.6 (Fig. 2B).

Considering that the criteria PA and ARR mainly focus on the sensitivity rather than specificity, we further calculated the ROC curves by using the methods provided in a previous study.^42^ The ability of FisherMP and other algorithms to recall real testing motifs was validated on 100 synthetic-binding peak files. We then plotted ROC curves and calculated the AUC for these seven algorithms (Fig. 2C and D). We found FisherMP can achieve a high true positive rate (TPR) of 80% while keeping a low false positive rate (FPR) of ∼7%. When the TPR of FisherMP is bigger than 90%, the FPR is still <12%. The AUC of FisherMP is 93.45%, which is the best among seven tested algorithms.

### 3.3. Super computational speed of FisherMP

**Figure 3:**
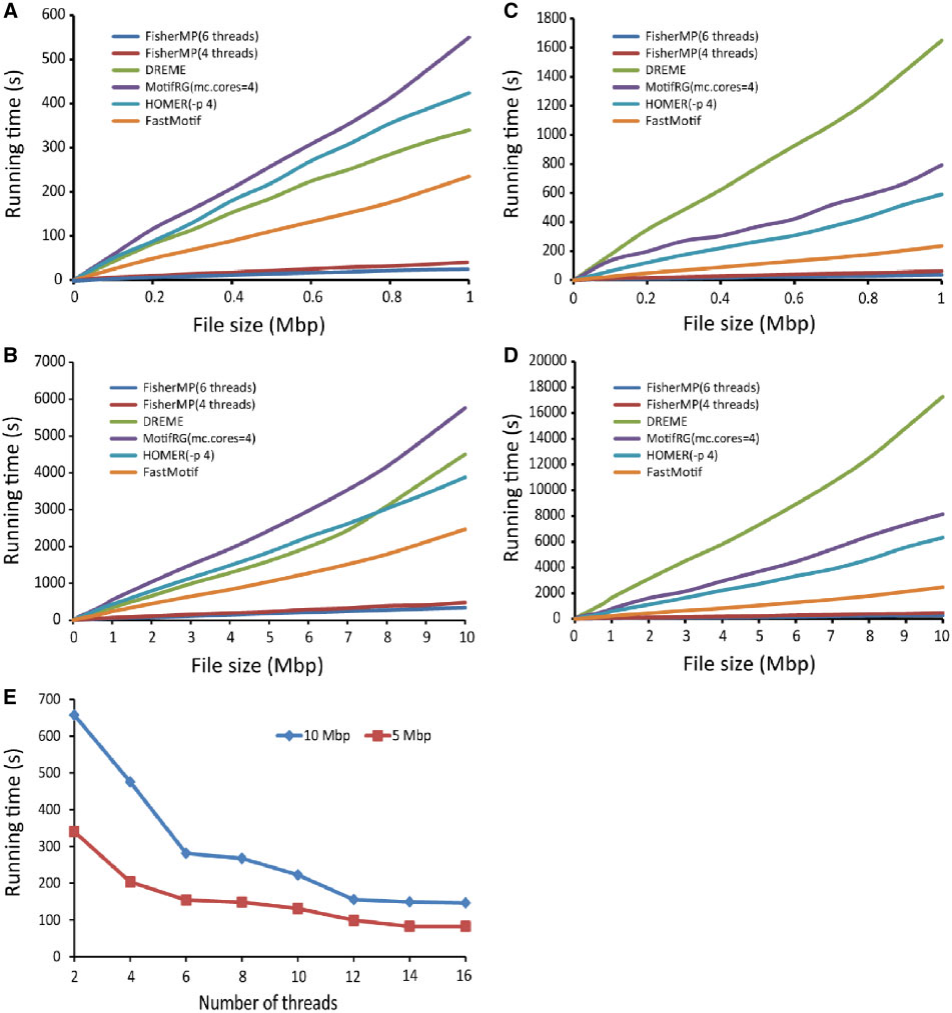
Comparative analysis of running time. Comparisons of running times on synthetic datasets (A) and (B) and human ENCODE datasets (C) and (D) with different sizes. (E) Running times of FisherMP on different number of threads.

The main goal of the FisherMP algorithm is to improve the computational speed of motif prediction. To test and prove its computational superiority, four relatively fast programmes were used for comparison. Four threads (or processes) were called in MotifRG and HOMER. For each dataset size (the length of each sequence is 1,000 bp), the average running time of a programme on 10 synthetic datasets of the size was obtained. As shown in Fig. 3A and B, FisherMP was the fastest among the five programmes if it also called four threads. In particular, for a big file of 10 Mb, FisherMP took only 300–400 s, while each of the other algorithms took thousands of seconds. For real ENCODE ChIP-binding peak data, as shown in Fig. 3C and D, FisherMP was also the fastest, and the running times had no significant change between real data and synthetic data. Note that the other algorithms were more time-consuming in the real data than in the synthetic data because each synthetic dataset was only embedded in one motif while the number of real motifs in a real dataset is unknown.

Because the design of FisherMP is fully parallelized, the algorithm will be faster if more threads are called. As with our previous experiments, the lengths of motifs searched by FisherMP were set with 6 different lengths (from 5 to 10), and the number of output motifs was set to 10. By changing the number of threads, the running time curve of FisherMP can be obtained. In order to see how efficient FisherMP was with the number of threads increasing, only the real ENCODE datasets with two big sizes (5 and 10 Mb size) were used to plot two running time curves, respectively (Fig. 3E). When the number of threads was set higher than 6, the running time of FisherMP was not severely affected. This is because FisherMP was executed with six different motif lengths in the experiments, and one motif length corresponding to one thread is the best parallel scheme.

### 3.4. Detecting combinatorial motifs

In mammalian genomes, gene expressions are widely controlled by combinatorial TFs but rarely by a single TF. From a ChIP-seq dataset of a TF, current motif-finding tools can usually find the binding peaks for a TF but can’t output the potential combinatorial TF motifs of its co-player TFs. One may overlap two sets of TF ChIP-seq data to get the co-occurrence of two TFs. However, there are more than 2,000 TFs encoded in the human genome and most of them have no antibodies, and thus no ChIP-seq data available. Considering that combinatorial *c*is-regulatory TFs in mammalian genomes typically exhibit their binding sites within the same genomic proximity, we hypothesized that it should be possible to discover the co-players and their potential combinatorial motifs for a targeted TF from its ChIP-seq by identifying over-represented combinations of sequence motifs that occur together in the ChIP-seq peaks. To this aim, we further extended our method to detect the combinatorial motifs by using a Fisher combined probability test of the *P*-values of single motifs.

We tested our strategy for the known TF combinations that were downloaded from TcoF database^40^ (latest version, March 2018). In our 51 selected TF ChIP-seq datasets, 21 TFs have known combinatorial motifs including 30 combinatorial motif pairs among them that belong to 4 cell lines, K562, GM12878, HepG2 and Hela-S3, where GM12878 has the most abundant resource of TF ChIP-seq in the ENCODE project database. For each of the 21 TFs, FisherMP was run on the peaks of its ChIP-seq datasets to detect whether the motifs of the TF and its cooperative TFs can be found in pairs with high confidence. We found that, among 24 of 30 datasets (24/30 = 80%), the true motif was top one ranked (Table 1). Of the remaining six datasets, two had the true motif ranked in the second position, three had the true motif ranked in the third position, and one had the true motif ranked in the sixth position. In total, the average rank of these TF motifs was 1.43 (s.d. = 1.07). We then checked the motifs for their cooperative TFs and found 25 of them successfully detected co-players (25/30 ≍ 83.33%). These 25 paired TFs were ranked very high (5.08 ± 5.33) and their combined *P*-values are very significant (*P*-values ≤ 1.35e-59). Considering that the binding sites and co-binding partners of each TF can be tissue-specific, we further checked the results of the same TF on two cell lines and found that the significance (*P*-value) of cooperative TFs are different between cells. For example, we can detect the cooperative pair of CEBPB and MYC from CEBPB ChIP-seq of K562 cell line but not from the CEBPB ChIP-seq data of GM12878 and HepG2 cell line respectively. Furthermore, we also observed the asymmetric ranking for a cooperative TF pair for a same cell line. For example, when checked the SRF ChIP-seq of K562, we can find the motifs of its cooperative TF, SP1, to be ranked in 10 (*P*-value 2.30e-82). However, SRF motifs can’t be detected by using SP1 ChIP-seq of K562. These results demonstrated that the TFs preferentially collaborate with different TFs for implementing cellular functions in different cells.

**Table 1:** Predicting 30 combinational motif pairs

ChIP-ed TF (TF A)	Cooperative TF (TF B)	Cell line	Motif A rank	Motif B rank	Motif A + B rank	*P*-value of motif A + B
NRF1	MAFF	K562	1	4	2	4.39e-246
IRF4	SPI1	GM12878	1	4	1	4.51e-325
E2F1	SP1	HeLa-S3	1	4	3	1.05e-248
CEBPB	HSF1	HepG2	1	5	4	1.38e-310
STAT1	HSF1	K562	3	2	2	2.49e-109
NR2C2	HNF4A	HepG2	6	6	1	0
STAT1	IRF1	K562	3	1	1	8.68e-120
BRCA1	STAT1	GM12878	1	4	1	4.96e-127
E2F4	BRCA1	GM12878	3	4	8	2.77e-183
NFYA	BRCA1	GM12878	1	—	—	—
BRCA1	SP1	GM12878	1	5	8	1.35e-59
NFYB	MYC	GM12878	1	—	—	—
GATA1/2	TAL1	K562	1	3	2	4.94e-324
GATA1	SP1	K562	1	10	17	2.81e-199
GATA2	SPI1	K562	1	4	3	9.87e-324
NFYA	ELF1	GM12878	1	6	2	1.65e-179
NFYA	SPI1	GM12878	1	9	11	1.44e-110
NFYA	SRF	GM12878	1	1	1	0
ELF1	SP1	GM12878	1	3	1	1.35e-325
EGR1	CEBPB	K562	1	—	—	—
EGR1	SP1	GM12878	1	1	1	0
SPI1	CEBPB	GM12878	1	10	14	4.37e-311
SRF	CEBPB	HepG2	1	—	—	—
CEBPB	SP1	K562	2	10	13	3.52e-245
CEBPB	MYC	K562	2	1	3	7.67e-294
SP1	TAL1	K562	1	11	16	3.55e-117
CTCF	YY1	GM12878	1	2	1	0
SRF	SP1	K562	1	4	10	2.3e-82
MEF2C	SP1	GM12878	1	—	—	—
MYC	SP1	GM12878	1	2	1	1.4e-244

‘—’ means out of detection.

We performed FisherMP on ER alpha ChIP-seq data of MCF-7 cell line and outputted top 10 ranked TFs with high significance (*P*-value < 1.0e-100, Supplementary Table S3). To characterize if these TFs are potential cooperative factors with ER alpha in MCF7-7 cell, we searched the publications in the PubMed database and found eight of them have been reported to be correlated with ER alpha in MCF-7 for regulating gene expressions. Additionally, many research papers had reported that the SP1, ZNF384, TP53 and TFAP2C are important cooperative players with ER alpha in breast cancer development.^43–46^ For example, SP1 is essential for the full transcriptional activity of ER alpha and their interactions will control the transcription of IGF-IQ gene whose dysregulated expression have pathologic consequences with relevance in breast cancer aetiology.^43^^,^^47^ Recent research revealed that ZNF384 can stimulate MCF-7 breast cancer growth by regulating cell cycle and metastasis-related genes via an ER alpha dependent pathway.^46^ These results demonstrated that our programme can well predict the cooperative factors even for tissue-specific TF.

### 3.5. CTCF and YY1 are co-players in human X chromosome

To further demonstrate the ability of FisherMP for finding true TF combinations, we performed integrative analysis of CTCF and YY1 for chromosome X in the GM12878 cell line. CTCF is a ubiquitously expressed protein with 11 zinc finger DNA-binding domains, and it is involved in the transcriptional regulation of many genes.^48^ Acting as a transcriptional activator, repressor, and insulator, it binds to tens of thousands of genomic sites and can interact with a plethora of other TFs.^49^ The divergent functions of CTCF and abundance of its cooperative TFs make it difficult to detect a specific cooperative TF. Among these cooperative TFs, YY1 was reported as a cofactor of CTCF for the chromosome X binary switch.^50^ Here we tested whether the motifs of CTCF and its co-factor YY1 can be found by these motif-finding tools in chromosome X. To this end, the binding peaks of CTCF in chromosome X (total 140,569 peak sequences) were isolated from the CTCF’s 99 ChIP-seq datasets and merged into a new file which contained 4471 unique binding peaks. After running FisherMP, XXmotif, MotifRG, DECOD, DREME, HOMER, and FastMotif on the new file, we found that FisherMP had the best performance. The top two output results of FisherMP correspond to the motifs of CTCF and YY1 (Table 2). Meanwhile, among the motifs output by the six motif-finding tools, FisherMP had the best fit with the two known motifs of CTCF and YY1, respectively. XXmotif and FastMotif could not output any motif similar to YY1’s directly. Even though the results of MotifRG, DREME, HOMER, and DECOD contain some motifs similar to YY1’s, their predicted YY1 motifs had lower ranking and lower similarity than FisherMP predictions. In addition, among the 4,471 peak sequences FisherMP found that 2584 sequences contain CTCF’s-binding sites and 2353 sequences contain the binding sites of both CTCF and YY1. This overlap was found to be very significant (*P*-value 8.02e-334, hypergeometric testing, one-sided), indicating that YY1 and CTCF work together on the X chromosome. We also performed the detections of the seven motif-finding tools on CTCF ChIP-seq of GM12878 cell line by using all chromosomes as a control. As shown in the Table 2, these tools, excepting FisherMP and DREME, are failed to find the YY1’s motifs in a genome wide way. Although MotifRG, HOMER, and DECOD can output suspected motifs of YY1 for the ChrX, they failed to detect the YY1 motifs by using all chromosomes.

**Table 2:** Comparisons of CTCF and YY1’s motifs output on ChrX and all chromosomes by seven motif-finding tools, respectively

Tools	Chr	Top 1 predicted motif	Predicted YY1’s motif	Rank of YY1 motif
FisherMP	ChrX	AG(g/a)(g/t/a)GGC	(g/a/c)CCAT	2
	All	GG(g/c)CAG(a/t/g)G	CCA(c/t)CT(a/c/t)	4
XXmotif	ChrX	(c/g/t)(a/g/t)(c/g)(c/t)GCC(a/c)(c/t)CT(a/g/t)(c/g)TGG	—	—
	All	(a/g/t)(c/g)(c/t)GC(c/a)(a/c)(c/t)CT(a/g/t)(c/g)TGG(g/a)	—	—
MotifRG	ChrX	C(c/g)(c/t)AGG(g/t)GGC	GCCATNTT	6
	All	C(c/g)(c/t)AG(c/a/g)(g/t)GGC	—	—
DREME	ChrX	AG(g/a)TGGC(g/a)	C(a/t)CCATCT	28
	All	AG(g/a)(g/t)GGC(a/g)	C(t/a)CCATCT	62
HOMER	ChrX	C(A/c/t)(c/g/t)C(t/a/c)NN(t/a)GG	TTT(c/g)CAT	20
	All	C(c/a)(c/g)(t/c)AG(g/a)(g/t)G	—	—
DECOD	ChrX	(C/g)(a/t)G(c/g)C(a/t)(g/c)(g/c)(a/t)G	(c/g)C(a/t)(t/g/c)(t/g/c)(c/g/t)(c/t)C(a/t)(g/c/t)	12
	All	(c/g)C(a/c/t)(c/g/t)C(t/a)G(g/c)(t/a)G	—	—
FastMotif	ChrX	(t/a)AGG(t/a)GGC(g/a)	—	—
	All	C(c/a/t)CAGC	—	—

‘—’ means out of detection.

**Figure 4:**
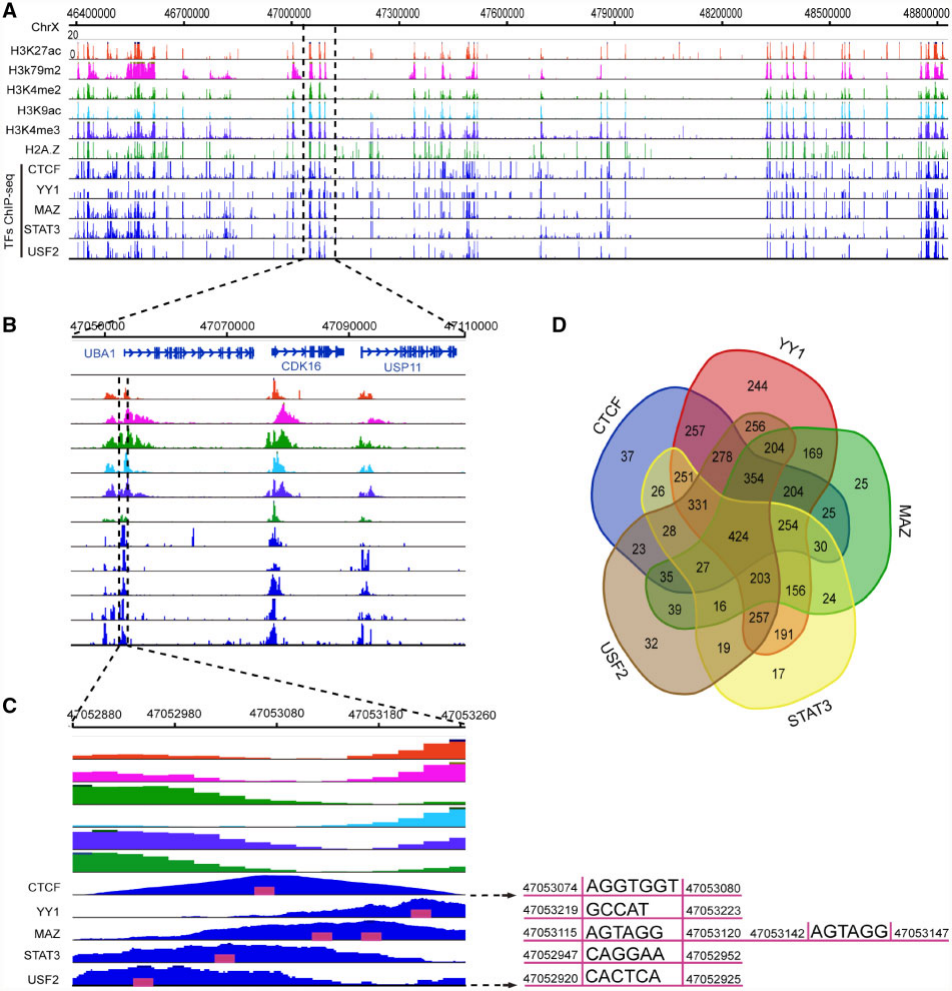
Integrative analysis of CTCF and combinatorial TFs on chrX. (A) The co-localizations of TF-binding peaks and histone modifications on a ∼2.4 Mb region of chrX. (B) A zoom-in region of three genes. (C) A zoom-in region shows the complementary signals of TF-binding peaks and histone modifications. The right side shows the DNA motifs and their coordinates of five TFs. (D) Venn graph shows the genomic overlapping of five TFs among CTCF-binding peaks.

We then performed a systematic analysis of all combinatorial motifs outputted in top 10 ranked results to check if they belonged to potential combinatorial TFs. Indeed, these motifs were found to be very significant and their consensus sequences were highly similar to the sequences provided in the JASPAR database (Supplementary Table S4). Among these top ten motifs, we aligned the binding peaks of YY1, MAZ, STAT3, and USF2 that had ChIP-seq data available in the ENCODE database. We found that the paired binding peaks were highly correlated to each other (Pearson correlation 0.71 ± 0.16). To understand the high correlations of these TFs and their potential functions, we illustrate an integrative analysis of a 2.4 Mb region (ChrX: 46400000-48800000) that displays TF motifs, binding peaks, and histone modifications. It is clearly shown that the TFs-binding peaks are not only highly coordinated with each other, but that they are also correlated to the histone modifications H3K27ac, H3K79me2, H3K4me2, H3K9ac, H3K4me3, and H2A.Z (Fig. 4A). Since these histone marks are usually located at active chromosomal regions, these motifs may collaborate with CTCF for gene regulation. In fact, the peaks of histone modifications and TF-binding peaks are mainly observed at gene promoter regions (Fig. 4B). Meanwhile, the TF-binding peaks are located within a ∼200 bp gap of histone modifications, suggesting that the histone proteins had been removed in order to facilitate TF binding (Fig. 4C). By searching the chromosomal sequence of this TF-binding region (400 bp, ChrX: 47052880-47053260), we successfully detected the motifs for these TFs (Fig. 4C, right side). For each TF, we further scanned 4471 CTCF peaks located on chromosome X to detect peaks that included its motifs. In total, we found 2,584, 4,033, 2,189, 2,254 and 2,526 peaks including CTCF, YY1, MAZ, STAT3 and USF2 motifs respectively. We observed they are highly overlapped (Fig. 4D) and their paired overlapping are significant (*P*-value < 0.05, hypergeometric testing, one-sided. Supplementary Table S5). We further tested that if the CTCF motifs can be detected from the YY1, MAZ, STAT3 and USF2 ChIP-seq data. As shown in Table 3, we found that CTCF motifs were ranked in top 10 in YY1, MAZ and STAT3 ChIP-seq data. However they are not top ranked in USF2 ChIP-seq data (rank of 21). These results were confirmed on all chromosomes, suggesting CTCF could be a major partner of YY1, MAZ and STAT3 in the whole genome. Meanwhile, CTCF might be a major partner of USF2 in the X chromosome but not in the whole genome. Taken together, the mutual analysis of these 5 TFs supports their genomic proximity as well as their co-occurrence with histone modifications, strongly suggesting that they are cooperative regulators of gene expression.

**Table 3:** Detect CTCF motifs from YY1, MAZ, STAT3 and USF2 ChIP-seq of GM12878 cell line

TF	Chr	Predicted CTCF motif	Rank of CTCF motif
YY1	ChrX	GA(a/g)GG	7
	All	A(g/a)ATGG(c/a/t)	2
MAZ	ChrX	GAGG(g/a)G	6
	All	AGGNGG	3
STAT3	ChrX	GGNGGA(a/g)	10
	All	GGAA(g/a/c)(t/a/g)G	4
USF2	ChrX	GG(g/a/t)GGA	2
	All	AGGAG(g/a)	21

FisherMP was run ChrX and all chromosomes (All), respectively.

## 4. Discussion

Finding the binding motifs of TFs and their combinations is a long standing problem in computational biology. Since it has been proved to be a NP-hard problem,^51^ suggesting there is no polynomial time algorithm to find the exact solution, the existing methods are all based on the balance of the precision and running speed. Many heuristic strategies have been used in these methods, but they are still quite time consuming on large scale ChIP-seq datasets. By fully using parallel computation technology, FisherMP is designed as an ultra-fast programme for discriminatively finding motifs on large-size ChIP-seq datasets while keeping relatively high precision. Actually, FisherMP is faster than most of other algorithms even when a single thread is called, since it is designed to avoid multiple nested loops and iteratively updating PWMs. FisherMP merges motifs and removes redundancies quickly (without complex iterations) by calculating motif similarity to reduce computing time. Furthermore, FisherMP identifies relatively more true motifs of the corresponding TFs from all the ChIP datasets of a species than the other tools, and has the additional capability of recovering many motifs of their co-factors in the meantime. A useful feature of the software is that it computes sequence indices containing all *cis*-regulatory elements of the motif and stores the information in a hash map. This enables FisherMP to output all important information including motif position, its PFM, PICs and PWM, which is convenient for researchers investigating motif profiles or performing module analyses. For example, the PFM and PWM outputted by our pipeline can be directly used to generate motif logos^52^ or calculate the binding energy.^53^ In genome-wide predicting *cis*-regulatory modules from a large number of ChIP-seq datasets, PWMs can be firstly used for scanning the motif positions among sequences to find homotypic or heterotypic clusters of binding sites for any combination of TFs.^30^

Although large scale validations on ENCODE ChIP-seq datasets demonstrate that FisherMP has exceptional performance in finding motifs, there is always room to improve precision and running speed. Also, although the majority of known motif profiles have lengths no >10 bp, we noticed that some longer KK motifs exist. The default parameters of FisherMP will not identify the full length of long KK motifs since the single motif length is defaulted as 10 in the pipeline. Users are suggested to extend the search range of motif lengths if they want to detect longer motifs. Furthermore, there is little accelerating room if the number of threads is >12, because the optimal number of threads is 2(*k*_max_–*k*_min_+1) = 12 to construct two hash maps in the first ‘fork-join’ structure of FisherMP. The default number of threads in FisherMP is set to *k*_max_–*k*_min_+1 if the minimum and maximum motif lengths are set to *k*_min_ and *k*_max_.

Finding combinatorial motifs is extremely important in real biological applications since gene expressions are widely controlled by combinatorial TFs. We had applied FisherMP on 30 known TF combinations provided by TcoF database^40^ and 25 pairs were successfully recalled, achieving a high precision of 83.33%. A detailed analysis of CTCF and YY1 shows their motifs are highly correlated in chrX. Besides CTCF and YY1, we also analysed the top ranked motifs of STAT3, MAZ and USP2, which all were clearly supported by the binding peaks from independent ChIP-seq data from the ENCODE project. Furthermore, these TF-binding peaks and their DNA motifs were also observed to be correlated with multiple histone modifications, exhibiting clusters of regulatory factors at gene promoter regions. In general, this integrative analysis of DNA motifs, binding peaks, histone modifications, and RNA-seq results can be performed for detecting not only active TFs related to gene expressions but also regulatory modules of TFs among divergent cells. In summary, these computational results and integrative analysis demonstrate that FisherMP is ultrafast and highly precise for detecting combinatorial motifs. We believe that FisherMP can help systematically investigate the mechanisms how TFs work collaboratively and coordinately in gene regulation.

## Data availability

FisherMP was parallelized with OpenMP and coded in C++ for achieving not only fast speed, but also the installation convenience. FisherMP is very easy to install since OpenMP is a standard library in the GCC and no additional plug-in for parallel programming is required. The C++ source code of FisherMP is publicly available at https://github.com/shaoqiangzhang/fishermp.

## Supplementary Material

dsz004_Supplementary_File
