# Supplementary material for: FisherMP: fully parallel algorithm for detecting combinatorial motifs from large ChIP-seq datasets
Source: DNA Res. 2019 Apr 8;26(3):231–42. doi: 10.1093/dnares/dsz004 (PMC6589551; doi:10.1093/dnares/dsz004)
Supplement: dsz004_Supplementary_File [file dsz004_supplementary_file.doc]

**Supplementary File1**

**FisherMP: Fully parallel algorithm for detecting combinatorial motifs from large ChIP-seq datasets**

Shaoqiang Zhang^1*^, Ying Liang^1^, Xiangyun Wang^1^, Zhengchang Su^1,2^, and Yong Chen^3*^

*^1^College of Computer and Information Engineering, Tianjin Normal University, Tianjin 300387, China*

^2^Department of Bioinformatics and Genomics, the University of North Carolina at Charlotte, NC 28223, USA

^3^Department of Biological Sciences, Center for Systems Biology, the University of Texas at Dallas, Richardson, TX 75080, USA

*To whom correspondence should be addressed. Yong Chen, Tel: +1 972 883 2528; Fax: +1 972 883 4551; Email: yong.chen1@utdallas.edu Correspondence may also be addressed to Shaoqiang Zhang. Tel: +86 18649006931; Email: zhangshaoqiang@tjnu.edu.cn

**Supplementary Sections**

1. **The approximation formula of p-values of Fisher’s exact test (Table S1)**
2. **The SPIC metric formula for calculating similarity between two motifs**
3. **Fisher combined method for calculating the p-value of combinational motifs**
4. **Figure S1 Informative analysis of core motifs in JASPAR database.**
5. **The used datasets from ENCODE project (Table S2)**
6. **Significant paired motifs detected from ER alpha ChIP-seq of MCF-7 (Table S3)**
7. **Significant paired motifs detected from chrX of CTCF ChIP-seq (Table S4,S5)**
8. **The commands of selected motif finding programs**
9. **The approximation formula of p-values of Fisher’s exact test:**

To test the significance that a motif is observed in a sequence set, we first construct the 2×2 contingency table.

**Table S1**: Contingency table of a motif that observed in a sequence set

| **Motif (or Kmer) Information** | **Foreground set** | **Background set** | **Row total** |
| --- | --- | --- | --- |
| # Sequences with the motif (or kmer) | *a* | *b* | *a+b* |
| #Sequences without the motif (or kmer) | *c* | *d* | *c+d* |
| Column total | *a+c* | *b+d* | *a+b+c+d* |

We then calculate the p-value $p=\frac{C_{a+b}^{a}C_{c+d}^{c}}{C_{a+b+c+d}^{a+c}}$of a Fisher’s exact test. We rewrite it as the following equivalent formulation:

$$p=e^{\ln p}=e^{\ln C_{a+b}^{a}+\ln C_{c+d}^{c}-\ln C_{a+b+c+d}^{a+c}}$$

Based on the Stirling formula , each combinatorial number can be approximated as

$$C_{n}^{k}=\frac{n!}{k!(n-k)!}\approx\frac{\sqrt{2\pi n}\left(\frac{n}{e}\right)^{n}}{\sqrt{2\pi k}\left(\frac{k}{e}\right)^{k}\sqrt{2\pi(n-k)}\left(\frac{n-k}{e}\right)^{n-k}}$$

$$=\frac{\sqrt{n}\left(n\right)^{n}}{\sqrt{2\pi k(n-k)}\left(k\right)^{k}\left(n-k\right)^{n-k}}$$

$$=\frac{n^{n+\frac{1}{2}}}{\sqrt{2\pi}\cdot k^{k+\frac{1}{2}}\left(n-k\right)^{n-k+\frac{1}{2}}}$$

Therefore, the logarithm of each combinatorial number can be approximated as

$$\ln C_{n}^{k}\approx(n+\frac{1}{2})\ln n-\frac{1}{2}\ln(2\pi)-(k+\frac{1}{2})\ln k-(n-k+\frac{1}{2})\log(n-k)$$

1. **The SPIC metric formula for calculating similarity between two motifs**

In our previous study, we had defined SPIC, a novel metric for calculating motif similarity . Here, we use the alignment-free SPIC or the SPIC with ungapped alignments to calculate the similarity between two motifs.

For a motif *M* of length *l*, its position weight matrix (PWM) is defined as

$w(b,i)=\log_{2}\frac{p\left(b,i\right)}{p\left(b\right)}\approx\log_{2}\frac{\text{ }\frac{f_{b,i}+\frac{\sqrt{N}}{4}}{N+\sqrt{N}}\text{ }}{p(b)}$, $b\in\{A,C,G,T\}$

where represents raw count of nucleotide *b* in position *i*, *N* is the number of sequences in the motif, is a pseudocount (correction for small sample size), and *p*(*b*) is the background frequency of nucleotide *b*. For each position *i*, the information content (IC) is defined as

$$I(i,W)=\sum_{b\in\{A,C,G,T\}} p(b,i)\cdot w(b,i)$$

For two motifs *M*_1_ and *M*_2_ with the same length *l*, if given their position frequency matrices (PFMs) and$\left(p_{2}(b,i)\right)_{4\times l}$, their PWMs and, and their position IC vectors and, respectively, the alignment-free SPIC metric (A-score) is defined as

$$A\text{-}score(M_{1},M_{2})=\frac{\max\left\{\sum_{i=1}^{l} I(i,W_{1})\sum_{b} \left(p_{2}(b,i)\cdot w_{1}(b,i)\right),\sum_{i=1}^{l} I(i,W_{2})\sum_{b} \left(p_{1}(b,i)\cdot w_{2}(b,i)\right)\right\}}{\max\left\{\sum_{i=1}^{l} I(i,W_{1})\sum_{b} \left(p_{1}(b,i)\cdot w_{1}(b,i)\right),\sum_{i=1}^{l} I(i,W_{2})\sum_{b} \left(p_{2}(b,i)\cdot w_{2}(b,i)\right)\right\}}$$

For two motifs *M*_1_ and *M*_2_ with motif lengths *l*_1_ and *l*_2_ respectively, The SPIC metric with ungapped alignments (B-score) is defined as

$B\text{-}score(M_{1},M_{2})=\frac{\text{max}\left\{\max_{a\in A} \left[\sum_{a} I(i,W_{1})\sum_{b} \left(p_{2}(b,a(i))\cdot w_{1}(b,i)\right)\right],\max_{a\in A} \left[\sum_{a} I(i,W_{2})\sum_{b} \left(p_{1}(b,a(i))\cdot w_{2}(b,i)\right)\right]\right\}}{\max\left\{\sum_{i=1}^{l_{1}} I(i,W_{1})\sum_{b} \left(p_{1}(b,i)\cdot w_{1}(b,i)\right),\sum_{i=1}^{l_{2}} I(i,W_{2})\sum_{b} \left(p_{2}(b,i)\cdot w_{2}(b,i)\right)\right\}}$, where position *a*(*i*) of one motif is aligned with position *i* of another motif in an alignment *a*, and *A* is the set of all possible ungapped alignments.

1. **Fisher combined method for calculating the p-value of combinational motifs**

With the p-value of each individual motif available, we used Fisher method to combine p-values to calculate a new p-value for the combinational motifs. Supposing *k* is the number of motifs for combinative analysis from total *m* motifs, we use fisher’s method to combine p-values into one [test statistic](https://en.wikipedia.org/wiki/Test_statistic)$\chi^{2}$ by using the formula$\chi_{2k}^{2}\sim-2\sum_{i=1}^{k} \ln(p_{i})$, where *p_i_* is the p-value for the hypothesis test of motif *i*. When the p-values tend to be small, the test statistic  will be large, suggesting high probability that the combinational motifs would be true. When *k*=1, it is the single motif case. For each *k,* we calculated all the motif combinations and outputted the Fisher combined p-value. To avoid the extreme case that there are no interactions among these single motifs, FisherMP computes the combined p-value for the sets of motifs that surpass certain threshold intersection ratios. Otherwise it does not compute the p-value. To clarify, FisherMP requires that the size of sequence intersection in the foreground sequence set is greater than that in the background sequence set. Since we used a hash table to store the sequence set containing the sites of each predicted motif in FisherMP, we can obtain, for a set of predicted motifs, the collection of sequences shared among these motifs. For a set of predicted motifs {*M_1_, M_2_, …, M_t_*}, where each motif *M_i_* covers the set of foreground sequences *F_i_* and the set of background sequences *B_i_*, FisherMP requires $\text{|}\bigcap_{i=1}^{t} F_{i}\text{|}>\text{|}\bigcap_{i=1}^{t} B_{i}\text{|}$. Second, since the sequences with motifs of *k* collaborative TFs are usually highly overlapped with each other, FisherMP further requires that intersection enrichment of foreground sequences of any motif pair *M_i_* and *M_j_* to be less than a p-value threshold of 0.05, where intersection enrichment is calculated by the hypergeometric testing. Please note, after calculating the single motifs, FisherMP only kept those significant motifs (p-value<1.0e-06) for further detecting combinatorial motifs. This p-value threshold also helps to avoid such rare cases that there are no interactions of two motif sets if they are considered to be uniformly distributed. For the special case of calculating combined p-values for paired motifs (*k*=2), we also provided an alternative testing strategy by using Fisher’s exact test to calculate p-values. In detail, for two motifs *M*_1_ and *M*_2_, whose corresponding foreground sequence sets are *F*_1_ and *F*_2_, and background sets are *B*_1_ and *B*_2_, we calculated the p-value by using Fisher’s exact test.

1. **Figure S1**. Informative analysis of core motifs in JASPAR database. **A.** The distribution of low position information contents for all core motifs in JASPAR 2018. **B.** The distributions of motif similarity scores for all core motifs.


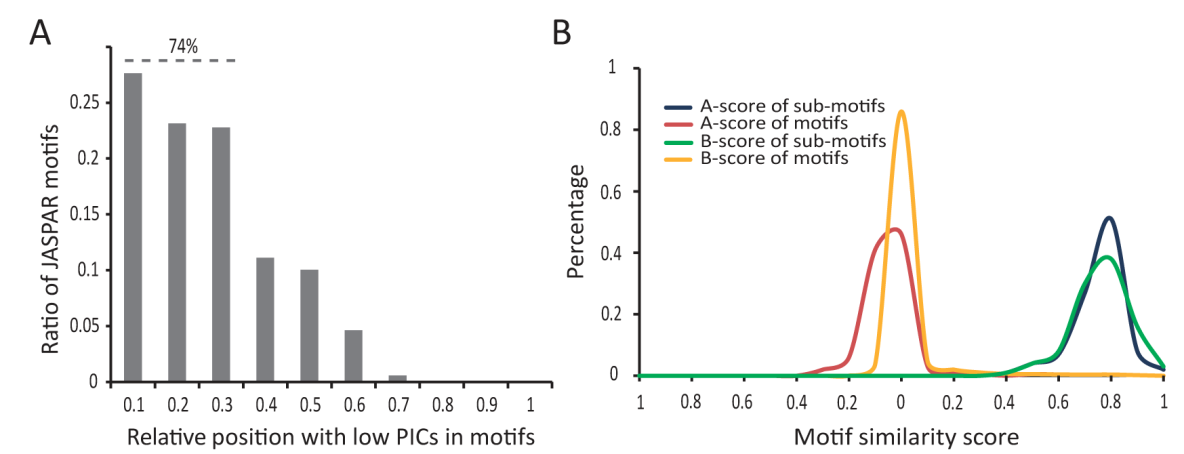


1. **The ENCODE datasets**

For validating the performance of our methods and identifying novel motifs for human important TFs, we used 51 TF’s known motifs and discovered (KK) motifs that were introduced by Kheradpour and Kellis . We downloaded these ENCODE TF ChIP-seq datasets from <http://compbio.mit.edu/encode-motifs/> and their uniform peaks from UCSC genome browser

(<http://genome.ucsc.edu/cgi-bin/hgFileUi?db=hg19&g=wgEncodeAwgTfbsUniform> ). In total, 350 ENCODE ChIP-Seq binding peak datasets were obtained to belong to 51 selected TFs.

**Table S2**：Statistic information of used datasets of ENCODE project

| **TFs** | **#datasets of ChIP-Seq peaks** | **#Known motifs** | **#Discovered motifs (KK motifs)** |
| --- | --- | --- | --- |
| ATF3 | 6 | 16 | 4 |
| BATF | 1 | 1 | 3 |
| BHLHE40 | 5 | 4 | 2 |
| BRCA1 | 4 | 2 | 1 |
| CEBPB | 10 | 10 | 2 |
| CTCF | 99 | 2 | 10 |
| E2F | 10 | 30 | 8 |
| EBF1 | 2 | 4 | 2 |
| EGR1 | 3 | 12 | 7 |
| ELF1 | 4 | 3 | 3 |
| EP300 | 12 | 1 | 10 |
| ESRRA | 1 | 11 | 4 |
| ETS | 3 | 18 | 9 |
| FOXA | 6 | 7 | 5 |
| GATA | 12 | 22 | 6 |
| HNF4 | 3 | 26 | 5 |
| HSF1 | 1 | 5 | 1 |
| IRF | 7 | 21 | 6 |
| MAF | 8 | 12 | 2 |
| MEF2 | 3 | 12 | 3 |
| MXI1 | 5 | 1 | 2 |
| MYC | 21 | 22 | 10 |
| NANOG | 1 | 1 | 4 |
| NFE2 | 2 | 2 | 4 |
| NFY | 6 | 6 | 1 |
| NR2C2 | 4 | 1 | 3 |
| NR3C1 | 5 | 18 | 6 |
| NRF1 | 5 | 2 | 3 |
| PAX5 | 4 | 5 | 5 |
| PBX3 | 1 | 1 | 3 |
| POU2F2 | 2 | 16 | 2 |
| POU5F1 | 1 | 5 | 2 |
| PRDM1 | 1 | 2 | 2 |
| REST | 12 | 4 | 10 |
| RFX5 | 5 | 9 | 3 |
| RXRA | 3 | 15 | 5 |
| SIX5 | 4 | 7 | 4 |
| SP1 | 4 | 9 | 3 |
| SPI1 | 3 | 4 | 3 |
| SREBP | 1 | 6 | 1 |
| SRF | 4 | 10 | 2 |
| STAT | 17 | 17 | 7 |
| TAL1 | 1 | 5 | 2 |
| TCF12 | 4 | 1 | 6 |
| TCF7L2 | 7 | 7 | 2 |
| TFAP2 | 2 | 22 | 2 |
| YY1 | 13 | 7 | 5 |
| ZBTB33 | 5 | 1 | 4 |
| ZBTB7A | 2 | 4 | 2 |
| ZEB1 | 1 | 5 | 1 |
| ZNF143 | 4 | 2 | 4 |

1. **Significant paired motifs detected from ER alpha ChIP-seq of MCF-7**

The binding peak file of ER in MCF-7 cell line was downloaded from GSE19013 (<https://www.ncbi.nlm.nih.gov/geo/query/acc.cgi?acc=GSE19013>). The matrix alignment tool (<http://jaspar.genereg.net/align>) was employed to compare the position frequency matrices (PFMs) of top 10 predicted motifs to all motifs of *Homo sapiens* in the JASPAR database. The top 10 predicted motifs and their best matches with percent scores greater than 0.7 in JASPAR database are listed in the Table S3.

**Table S3**：Top 10 ranked combinatorial motifs predicted from ER alpha ChIP-seq of MCF-7.

| **TFs** | **Motif logo** | **Motif logo in Jaspar** | **Rank** | **P-value** | **Combined P-value** | **PMID** |
| --- | --- | --- | --- | --- | --- | --- |
| SP1 | 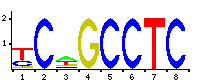 | 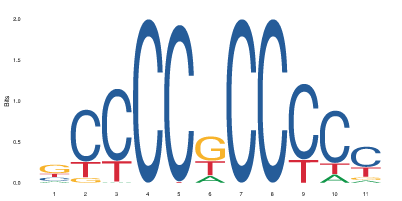 | 1 | 2.362e-349 | 3.53e-448 | 12429135  15111769  22407812 |
| ZNF384 | 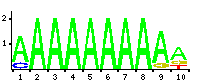 | 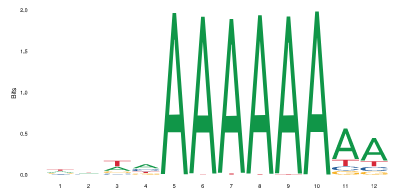 | 2 | 6.784e-323 | 4.56e-445 | 26407941  25849766 |
| TP53 | 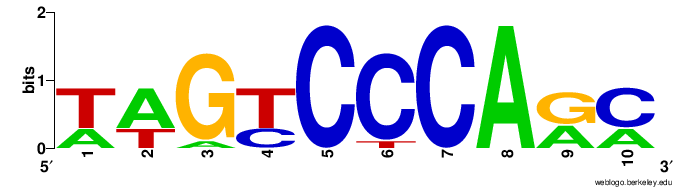 | 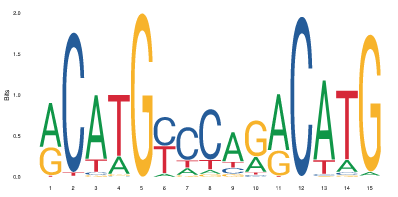 | 3 | 1.14e-315 | 2.21e-443 | 19351845  21191649  21737614 |
| EBF1 | 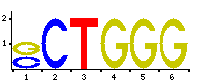 | 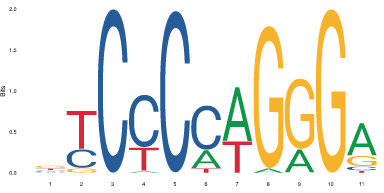 | 4 | 7.288e-313 | 4.56e-439 | 23951143  21235772 |
| TFAP2C | 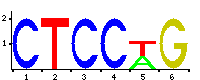 | 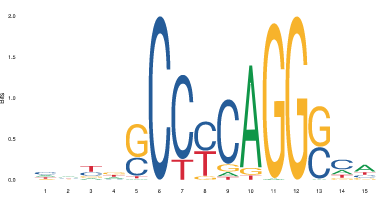 | 5 | 5.457e-311 | 6.75e-437 | 26160249  19458056  20629094 |
| TAL1 | 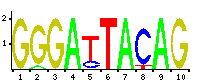 | 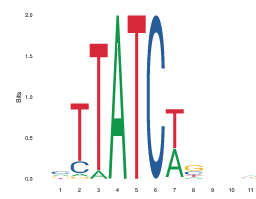 | 6 | 6.717e-309 | 1.68e-435 |  |
| TFAP2A | 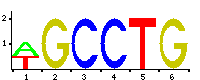 | 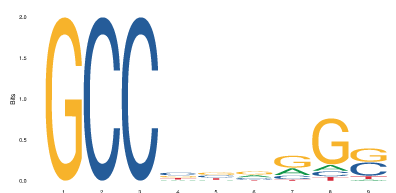 | 7 | 1.457e-297 | 5.34e-433 | 19458056 |
| ZBTB7B | 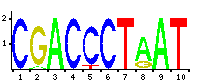 | 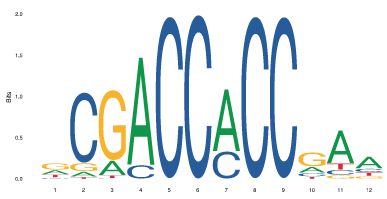 | 8 | 9.845e-231 | 2.52e-432 | 22976807  30265334 |
| PAX5 | 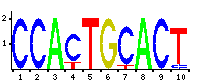 | 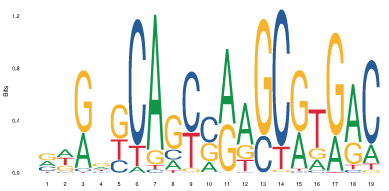 | 9 | 1.6e-203 | 2.86e-328 | 23636943  23950888 |
| MAFK | 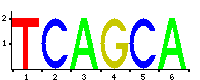 | 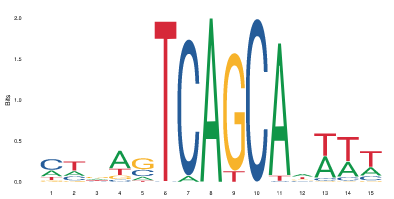 | 10 | 2.341e-193 | 4.65e-325 |  |

1. **Significant paired motifs detected from chrX of CTCF ChIP-seq**

The matrix alignment tool (<http://jaspar.genereg.net/align>) was employed to compare the position frequency matrices (PFMs) of top 10 predicted motifs to all motifs of *Homo sapiens* in the JASPAR database. The best matches with percent scores greater than 0.7 are listed in the Table S4.

**Table S4**：Top 10 ranked motifs paired with CTCF.

| **TFs** | **Motif logo** | **Motif logo in JASPAR** | **Rank** | **P-value** | **Combined P-value** |
| --- | --- | --- | --- | --- | --- |
| YY1 | 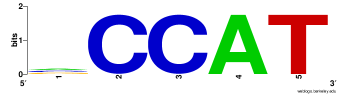 | 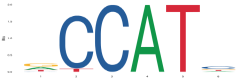 | 1 | 1.84e-214 | 6.65e-279 |
| FEV | 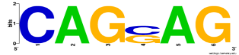 | 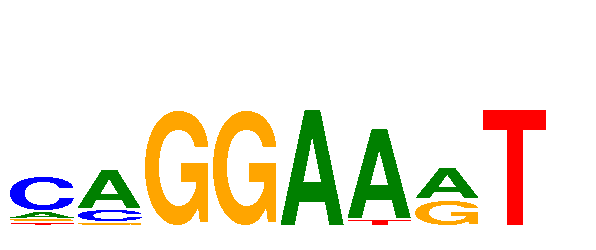 | 2 | 3.815e-213 | 5.14e-265 |
| STAT1 | 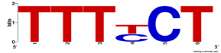 | 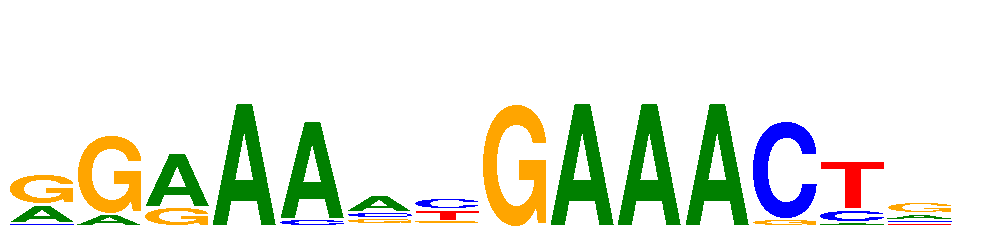 | 3 | 4.56e-210 | 3.48e-251 |
| MAZ | 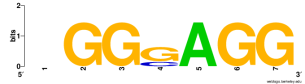 | 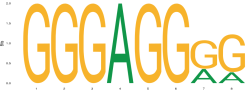 | 4 | 2.97e-197 | 8.49e-245 |
| STAT3 | 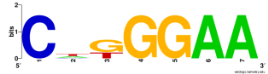 | 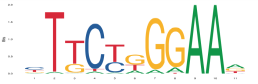 | 5 | 1.55e-191 | 2.88e-216 |
| NFIC | 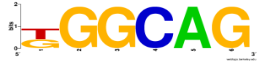 | 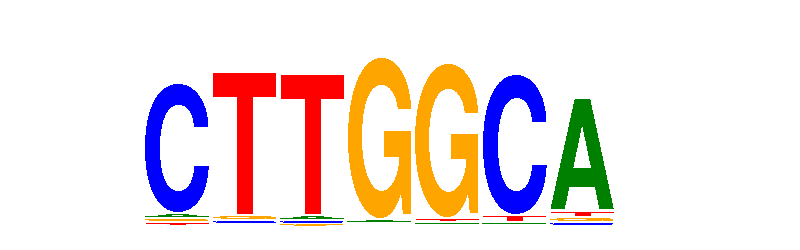 | 6 | 1.12e-189 | 1.07e-210 |
| USF2 | 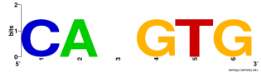 | 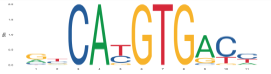 | 7 | 6.38e-188 | 2.26e-203 |
| STAT2 | 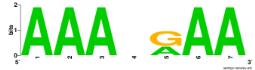 | 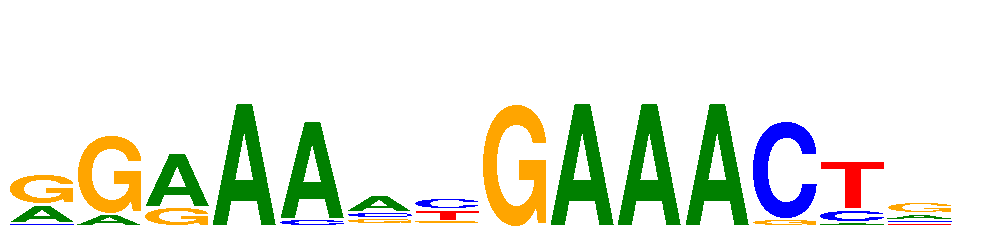 | 8 | 9.22e-177 | 1.64e-199 |
| KLF4 | 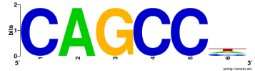 | 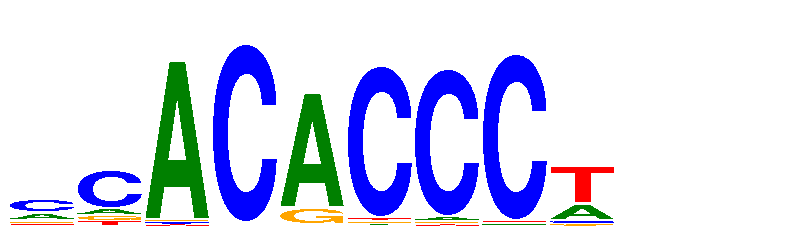 | 9 | 1.07e-171 | 1.37e-195 |
| CTCFL | 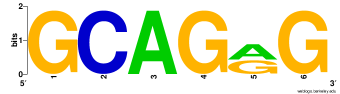 | 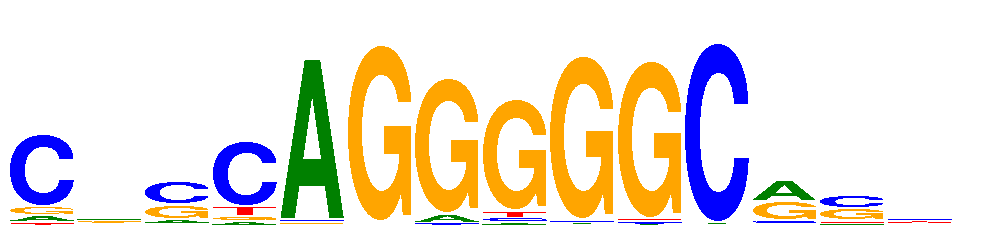 | 10 | 1.10e-159 | 2.37e-192 |

**Table S5：Statistical analysis of paired motifs among total 4471 peaks. The lower triangular area of the table shows the numbers of overlapping peaks of two motifs. The upper triangular area of the table shows the p-values of overlapping enrichment of two motifs that were calculated by using hypergeometric testing, one-side.**

|  | **CTCF:2584** | **YY1:4033** | **MAZ:2189** | **STAT3:2254** | **USF2:2526** |
| --- | --- | --- | --- | --- | --- |
| **CTCF: 2584** |  | P(x>2353)=8.02e-334 | P(x>1353)=5.9e-08 | P(x>1371)=1.99e-05 | P(x>1500)=7.79e-03 |
| **YY1: 4033** | 2353 |  | P(x>1968)=0.027 | P(x>2067)=3.39e-04 | P(x>2307)=2.34e-03 |
| **MAZ: 2189** | 1353 | 1968 |  | P(x>1134)=3.65e-02 | P(x>1302)=3.36e-10 |
| **STAT3: 2254** | 1371 | 2067 | 1134 |  | P(x>2353)=3.05e-02 |
| **USF2: 2526** | 1500 | 2307 | 1302 | 1305 |  |

1. **The commands of the selected motif finding programs**

To demonstrate the performance of our method, we compare it with other existing methods. For each method, the latest version of its software was downloaded and the input files were prepared according to their manual introduction. In brief, the foreground FASTA file was prepared and named as “input_file.fa”, and the background sequences for the “input_file.fa” was then produced by each of these programs in their default settings if available. Otherwise the background file “negative_file.fa” was created by shuffling the “input_file.fa”.

For each tool, motif lengths were set from 5 to 10, the number of output motifs was set 10, the number of threads (or processes) was set 4, both strands (i.e., each sequence and its reverse complement) were set to be searched, and the p-value was set 0.05 if these corresponding parameters exist. The detailed commands of each method are described as follows.

1. The command line of FisherMP : (C++ program)

./fishermp input_file.fa -m 5 -M 10 -n 10 -t 6 >output_file

./fishermp input_file.fa -m 5 -M 10 -n 10 -t 4 >output_file

1. The command line of DREME：(Python program)

./dreme -p input_file.fa -mink 5 -maxk 10 -m 10 -o output_dir

1. HOMER：(Perl program)

./findMotifs.pl input_file.fa fasta output_dir -len 5,6,7,8,9,10 -p 4 -basic

1. MotifRG: (R program)

findMotif(input_file.fa, category, weights = rep(1, length(input_file.fa)), start.width=5, min.cutoff=5, min.ratio=1.3, min.frac=0.01, both.strand=TRUE, flank=2, max.motif=10, mask=TRUE, other.data=NULL, start.nmer=NULL, enriched.only=F, n.bootstrap = 5, bootstrap.pvalue=0.1, is.parallel = TRUE, mc.cores = 4, min.info=10, max.width=10, discretize=TRUE)

1. XXmotif: (C++ program)

XXmotif out_dir input_file.fa --no-graphics --mops --revcomp

1. DECOD (Java program)

java -jar DECOD-20111024.jar -nogui -pos input_file.fa -neg negative_file.fa -w 10 -nmotif 10 -o output_file -strand both

1. FastMotif (Matlab program)

[pvalue, time]=FastMotif(input_file.fa, output_file)

Note that the “fastmotif.m” file was modified to set “output motif number=10”.

**References:**

1. Zhang, S., Zhou, X., Du, C. and Su, Z. (2013) SPIC: A novel similarity metric for comparing transcription factor binding site motifs based on information contents. *BMC systems biology*, **7 Suppl 2**, S14.

2. Kheradpour, P. and Kellis, M. (2014) Systematic discovery and characterization of regulatory motifs in ENCODE TF. *Nucleic Acids Res*, **42**, 2976-2987 LID - 2910.1093/nar/gkt1249 [doi].
